# Analysis of sleep patterns among clinical nurses: a latent profile and association rule mining approach

**DOI:** 10.1186/s12912-025-04026-4

**Published:** 2025-11-17

**Authors:** Ning Wei, Lulu Hu, Jian Li, Jianying Chu

**Affiliations:** 1 Department of Obstetrics and Gynaecology, Qilu Hospital of Shandong University, Jinan, 250012 China; 2 Department of Anaesthesia, Qilu Hospital of Shandong University, Jinan, 250012 China

**Keywords:** Latent profile analysis, Association rule mining, Clinical nurses, Sleep quality, Heterogeneity, Influencing factors

## Abstract

**Background:**

Traditional approaches to assessing sleep quality in clinical nurses often overlook population heterogeneity and the complex interplay of influencing factors. This study employs Latent Profile Analysis (LPA) and Association Rule Mining (ARM) to identify distinct sleep quality subgroups and uncover key factor combinations, thereby informing targeted intervention strategies.

**Methods:**

A total of 1,686 nurses from 123 hospitals in Shandong Province were recruited through multistage stratified sampling. LPA was used to classify participants based on seven sleep dimensions from the Pittsburgh Sleep Quality Index (PSQI), while ARM was applied to identify frequent itemsets of sleep disorder triggers. Key influencing factors were further examined using univariate analysis and multivariate logistic regression.

**Results:**

Three latent sleep profiles were identified: high (63.11%), moderate (34.10%), and low (2.79%) sleep quality. The low-sleep subgroup was characterized by higher proportions of being unmarried/divorced (42.55%), low monthly income (≤ 3,000 CNY, 42.55%), non-permanent employment (76.60%), and severe psychological distress (44.68%). In contrast, the high-sleep subgroup featured higher rates of being married (85.62%), moderate income (3,001–7,000 CNY, 73.03%), and low psychological distress (51.32%). Key determinants included marital status (OR = 2.153/2.252), income (OR = 9.098), employment type (OR = 1.475), and psychological state (OR = 0.060–0.555). ARM revealed distinct risk combinations: “low income + non-permanent employment” (lift = 3.895) for the low-sleep group; “married + moderate income + non-permanent employment + patient conflict” for the moderate group; and “high income + low psychological distress” buffering night-shift effects in the high-sleep group.

**Conclusion:**

By integrating LPA and ARM, this study reveals the multidimensional heterogeneity and interactive mechanisms underlying clinical nurses’ sleep quality. The findings support a stratified intervention framework combining institutional safeguards with precision strategies to enhance sleep health management in nursing populations.

## Background

In China’s current healthcare system, clinical nurses persistently face sleep health challenges due to rotating shift schedules. Under predominant three-shift (8-hour shifts) and two-shift (12-hour shifts) systems, nurses undertake 6–10 night shifts monthly [1]. This high-intensity, irregular work pattern renders nurses a high-risk population for sleep quality deterioration [2, 3]. A nationwide large-scale survey encompassing 274,123 nurses from 136 public hospitals across 31 provinces revealed that 46.87% of nurses averaged less than the WHO-recommended 7 h of daily sleep, with 27.63% reporting low subjective sleep satisfaction [4].

Sleep quality is not only critical to nurses’ individual health but also intrinsically linked to occupational risks. Evidence-based medical research demonstrates that chronic poor sleep quality significantly elevates cardiovascular disease risks [5], while correlating strongly with gastrointestinal dysfunction and cognitive impairment [6, 7]. Furthermore, fatigue induced by sleep deprivation increases the likelihood of medication errors, documentation lapses, and other decision-making failures, directly jeopardizing patient safety and care quality [8]. Thus, systematically investigating sleep quality determinants and enabling early identification of sleep disorder risks are pivotal for safeguarding nurses’ well-being and healthcare safety.

However, current methodologies in nurse sleep research exhibit notable limitations. Traditional scale-based assessments oversimplify multidimensional sleep issues into unidimensional continuous variables, obscuring population heterogeneity and masking critical subtype characteristics of sleep disorders. Conventional regression analyses fail to capture nonlinear interactions among multifactorial variables, thereby neglecting clinically meaningful attribute combination rules [9]. To address these gaps, this study establishes a dual-phase analytical framework: Latent Profile Analysis (LPA) identifies latent subgroups based on multidimensional sleep indicators, overcoming dimensional constraints of conventional classifications [10]; Association Rule Mining (ARM) employs the Apriori algorithm to decode frequent itemsets among physiological parameters, job characteristics, and environmental factors, revealing key pathogenic combinations of sleep disorders [11].

This research aims to achieve precise classification of clinical nurses’ sleep status, elucidate differential influencing factors across subgroups, and provide scientific foundations for developing targeted sleep intervention strategies.

## Study participants and methods

### Study participants

This study adopted a multistage stratified sampling method to select 1,686 nurses from general hospitals in Shandong Province between November 2021 and January 2022. The sampling process involved stratifying all prefecture-level cities in Shandong Province into three categories (high, medium, and low) based on their 2020 per capita GDP. From each category, one city was randomly selected: Qingdao (high GDP tier), Zaozhuang (medium GDP tier), and Dezhou (low GDP tier). In cities with only one municipal general hospital, that hospital was directly included in the survey. For cities with multiple hospitals, one general hospital was randomly chosen. Subsequently, three counties (or districts/county-level cities) were randomly selected from each sampled prefecture-level city, and one general hospital was randomly selected from each county, resulting in a total of 12 hospitals (3 municipal and 9 county-level). Within each hospital, three departments were randomly selected, and all night-shift nurses in those departments participated in the survey. The study protocol was approved by the Ethics Committee of Shandong University School of Public Health (Approval No. 20181219). It should be noted that the data collection period (November 2021 to January 2022) coincided with the COVID-19 pandemic in China. The heightened workload and psychological stress experienced by clinical nurses during this period may have elevated the overall levels of sleep disturbances and psychological distress observed in this study, thereby influencing the results [12, 13].

### Research instruments

#### Demographic questionnaire

A self-designed demographic questionnaire was developed through systematic literature review and adaptation of prior research tools. The questionnaire collected comprehensive information across 21 items, including gender, age, religious affiliation, highest educational attainment, marital status, average monthly income, number of children, years of nursing experience, years employed at the current institution, department affiliation, and average monthly night shifts.

#### Pittsburgh Sleep Quality Index (PSQI)

The PSQI, originally developed by Buysse et al. and adapted into Chinese by Liu Xianchen et al. [14], is widely used in healthcare populations. The scale comprises 18 self-rated items assessing seven dimensions: subjective sleep quality, sleep latency, sleep duration, sleep efficiency, sleep disturbances, use of hypnotic medications, and daytime dysfunction. Each dimension is scored from 0 to 3, with a total score ranging from 0 to 21. Higher scores indicate poorer sleep quality. In this study, the Cronbach’s α coefficient for the PSQI was 0.879, demonstrating good reliability.

#### Kessler psychological distress Scale-10 (K10)

The K10, developed by Kessler and Mroczek in 1994 and validated in Chinese by Zhou Chengchao et al. [15], measures psychological distress. The scale includes 10 items rated on a 5-point Likert scale (“1 = almost never” to “5 = always”), with total scores ranging from 10 to 50. Higher scores reflect greater psychological distress. The Cronbach’s α coefficient for the K10 in this study was 0.930, indicating high internal consistency.

#### Work-family conflict scale (WAFCS)

The WAFCS, developed by Grzywacz et al. [16], evaluates conflicts between work and family responsibilities. The 11-item scale consists of three subscales: work-to-family interference (4 items), family-to-work interference (4 items), and family-to-work facilitation (3 items). Responses are recorded on a 5-point Likert scale (“1 = strongly disagree” to “5 = strongly agree”). The Cronbach’s α coefficient for the WAFCS in this study was 0.947, confirming its reliability.

#### Perceived social support scale (PSS)

The PSS, originally developed by Zimet et al. and adapted into Chinese by Jiang Qianjin [17], assesses perceived social support. The scale contains 12 items rated on a 7-point Likert scale (“1 = strongly disagree” to “7 = strongly agree”), covering three dimensions: family support, friend support, and support from significant others. Total scores range from 12 to 84, with higher scores indicating stronger perceived social support. The Cronbach’s α coefficient for the PSS in this study was 0.956, demonstrating excellent reliability.

### Statistical analysis

Latent class analysis (LCA) was performed using Mplus 8.3 to classify participants’ sleep status. The modeling process began with a single-class model (C1), incrementally increasing the number of latent profiles. Model selection relied on goodness-of-fit and comparative tests, evaluated through the following criteria:1.Akaike Information Criterion (AIC), Bayesian Information Criterion (BIC), and sample-size-adjusted BIC (SaBIC), where lower values indicate superior model fit. 2.Entropy index to assess classification accuracy, with values ≥ 0.6 and ≥ 0.8 corresponding to classification accuracy rates exceeding 80% and 90%, respectively. 3.Lo-Mendell-Rubin adjusted likelihood ratio test (LMR) and Bootstrap likelihood ratio test (BLRT), where statistically significant P-values (< 0.05) indicated that the k-class model outperformed the (k-1)-class model.

Data entry and preliminary analyses were conducted in SPSS 25.0. The normality of continuous variables was assessed using the Shapiro-Wilk test and visual inspection of Q-Q plots. Given the large sample size (*n* = 1,686) and the central limit theorem, the use of t-tests and ANOVA was considered robust. Furthermore, due to mandatory fields in the electronic questionnaire, there were no missing data for the core variables used in this analysis. Categorical variables were summarized as frequencies and percentages (%), while continuous variables were reported as mean ± standard deviation. Given the exploratory nature of the numerous univariate group comparisons, the results should be interpreted with caution, and the primary focus is placed on the subsequent multivariate models and effect sizes. Group comparisons utilized t-tests and analysis of variance (ANOVA). Multivariate logistic regression identified factors influencing sleep classifications, with statistical significance set at *P* < 0.05.

Association rule mining for sleep status was implemented via the Apriori algorithm in SPSS Modeler 18.0. Categorical variables were analyzed using frequency and percentage (%). χ² tests in SPSS 25.0 evaluated antecedent-consequent relationships in the association rules, with a significance threshold of α = 0.05.

## Results

### Sociodemographic characteristics of participants

The study included 1,686 clinical nurses. The majority were female (95.67%, *n* = 1,613), with male nurses comprising 4.33% (*n* = 73). Age distribution showed the largest proportion (44.42%, *n* = 749) in the 30–39 age group, followed by 20–29-year-olds (36.36%, *n* = 613). A significant majority (96.74%) reported no religious affiliation. Education levels were predominantly bachelor’s degrees (79.83%, *n* = 1,346), and most participants were married (81.79%, *n* = 1,379). Regarding income, 43.71% (*n* = 737) earned between 3,001 and 5,000 CNY monthly. Over half (52.91%) had one child.

Occupational characteristics revealed that 31.55% had 6–10 years of nursing experience, while 48.10% worked in tertiary Grade A hospitals. Nearly half (46.80%) reported experiencing verbal or physical abuse from patients or their families. Psychologically, 37.96% exhibited low psychological distress, whereas 35.23% reported high work-family conflict. Detailed data are presented in Table 1.

**Table 1:** Sociodemographic information of study participants

Variable	Frequency	Percentage
Gender		
Male	73	4.33%
Female	1613	95.67%
Age		
20–29	613	36.36%
30–39	749	44.42%
40+	324	19.22%
Religious Belief		
No	1631	96.74%
Yes	55	3.26%
Highest Education Level		
Secondary Vocational School	22	1.30%
Junior College	285	16.90%
Bachelor’s Degree	1346	79.83%
Master’s Degree or Above	33	1.96%
Marital Status		
Unmarried	288	17.08%
Married	1379	81.79%
Others	19	1.13%
Average Monthly Income		
<3000 RMB	203	12.04%
3001–5000 RMB	737	43.71%
5001–7000 RMB	511	30.31%
7001–9000 RMB	175	10.38%
>9000 RMB	60	3.56%
Number of Children		
No Children	486	28.83%
One	892	52.91%
Two or More	308	18.27%
Years in Nursing Profession		
0–5 Years	452	26.81%
6–10 Years	532	31.55%
11–15 Years	276	16.37%
16–20 Years	173	10.26%
20 + Years	253	15.01%
Years in Current Institution		
0–5 Years	553	32.80%
6–10 Years	507	30.07%
11–15 Years	251	14.89%
16–20 Years	152	9.02%
20 + Years	223	13.23%
Department		
Internal Medicine	454	26.93%
Surgery	385	22.84%
Obstetrics and Gynecology	304	18.03%
Pediatrics	223	13.23%
Others	320	18.98%
Professional Title		
No Title	76	4.51%
Technician Level	214	12.69%
Junior Professional (Initial)	814	48.28%
Intermediate Level	499	29.60%
Associate Senior or Above	83	4.92%
Administrative Position		
No	1404	83.27%
Yes	282	16.73%
On-the-Job Staff Status		
No	1056	62.63%
Yes	630	37.37%
Hospital Level		
Second-Class B and Below	679	40.27%
Third-Class B	196	11.63%
Third-Class A	811	48.10%
Average Weekly Work Hours		
≤ 40 h	779	46.20%
41–50 h	679	40.27%
> 50 h	228	13.52%
Monthly Night Shifts		
≤ 7 Days	1174	69.63%
> 7 Days	512	30.37%
Perceived Respect from Patients		
Disrespected	96	5.69%
Neutral	725	43.00%
Respected	865	51.30%
Experienced Patient/Visitor Violence		
No	897	53.20%
Yes	789	46.80%
Current Work Environment		
Poor	355	21.06%
Moderate	844	50.06%
Good	487	28.88%
Medical Errors in Past Year		
No	1641	97.33%
Yes	45	2.67%
Seriously Considered Suicide		
Yes	155	9.19%
No	1531	90.81%
Psychological Distress		
Low Distress	640	37.96%
Moderate Distress	460	27.28%
High Distress	322	19.10%
Severe Distress	264	15.66%
Work-Family Conflict		
Low Conflict	282	16.73%
Moderate Conflict	810	48.04%
High Conflict	594	35.23%
Social Support		
Low Support	82	4.86%
Moderate Support	594	35.23%
High Support	1010	59.91%

### Scores across sleep status dimensions

The mean scores for sleep quality and sleep efficiency were 0.11 and 0.53, respectively, while the remaining five sleep dimensions (sleep latency, sleep duration, sleep disturbances, use of hypnotic medications, and daytime dysfunction) had mean scores ranging between 1.0 and 1.2. Notably, the standard deviations for all dimensions exceeded half of their corresponding mean values, indicating substantial heterogeneity in participants’ responses across the seven sleep dimensions. Detailed results are presented in Table 2.

**Table 2:** Scores for dimensions of sleep quality

Dimension	Mean	Median	Std. Deviation	Minimum	Maximum
Sleep Quality	0.11	0	0.46	0	3
Sleep Latency	1.20	1	0.89	0	3
Sleep Duration	1.05	1	0.82	0	3
Sleep Efficiency	0.53	0	0.86	0	3
Sleep Disturbances	1.04	1	0.62	0	3
Use of Sleep Medications	1.09	1	1.08	0	3
Daytime Dysfunction	1.20	1	0.91	0	3

### Latent profile analysis of sleep status

**Fig. 1:**
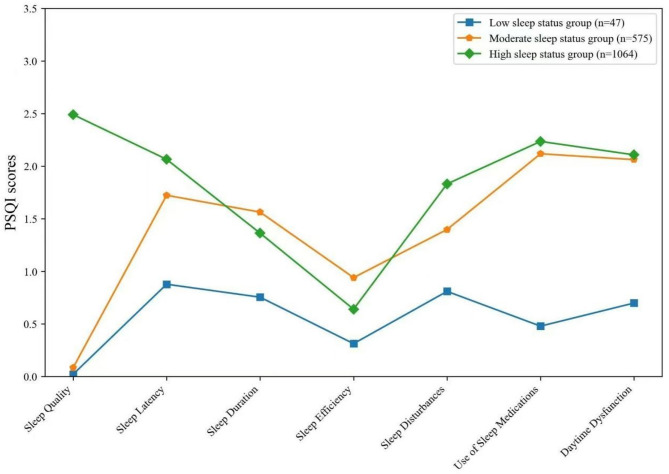
Classification of sleep status types based on latent profile

Starting with a single-profile model, the number of latent profiles was incrementally increased to identify the optimal model. As shown in the model fit indices (Table 3), the AIC, BIC, and SaBIC values decreased progressively with additional profiles. The three-profile model demonstrated an entropy value of 0.889, with both the Lo-Mendell-Rubin (LMR) and Bootstrap Likelihood Ratio Test (BLRT) yielding statistically significant P-values (*P* < 0.001). Although the four-profile model achieved a slightly higher entropy (0.893), its LMR P-value (0.084) lacked statistical significance. The five-profile model, despite having the highest entropy (0.926) and significant LMR/BLRT results (*P* < 0.001), included one profile with zero participants, rendering it impractical. Based on parsimony and interpretability, the three-profile model was selected as optimal. The three profiles comprised: Class 1 (*n* = 1,064, 63.11%*): Characterized by the highest overall sleep quality. Class 2 (*n* = 575, 34.10%*): Exhibiting moderate sleep quality. Class 3 (*n* = 47, 2.79%*): Demonstrating the poorest sleep quality. These groups were labeled as the High Sleep Status Group, Moderate Sleep Status Group, and Low Sleep Status Group, respectively. Mean scores for each class across the seven sleep dimensions are detailed in Table 4 and visualized in Fig. 1.

**Table 3:** Latent profile analysis (LPA) indicators of sleep quality

Classes	AIC	BIC	SaBIC	Entropy	LMR	BLRT	Class Counts/Proportions
1	27610.99	27687.01	27642.53				
2	25452.36	25571.82	25501.93	0.828	< 0.001	< 0.001	1087 (64.47%)/599 (35.53%)
3	23289.68	23452.58	23357.28	0.889	< 0.001	< 0.001	1064 (63.11%)/575 (34.10%)/47 (2.79%)
4	22512.82	22719.16	22598.44	0.893	0.084	< 0.001	949 (56.29%)/432 (25.62%)/258 (15.30%)/47 (2.79%)
5	20744.58	20994.36	20848.23	0.926	< 0.001	< 0.001	0 (0.00%)/1036 (61.45%)/47 (2.79%)/68 (4.03%)/535 (31.73%)

**Table 4:** Univariate analysis of sleep quality latent profiles

Variable	High Sleep Status Group (*n* = 1064)	Moderate Sleep Status Group(*n* = 575)	Low Sleep Status Group(*n* = 47)	Statistic	*P*-Value
Gender				4.868	0.088
Male	46(4.32%)	22(3.83%)	5(10.64%)		
Female	1018(95.68%)	553(96.17%)	42(89.36%)		
Age				9.640b	0.047
20–29	382(35.9%)	205(35.65%)	26(55.32%)		
30–39	468(43.98%)	268(46.61%)	13(27.66%)		
40+	214(20.11%)	102(17.74%)	8(17.02%)		
Religious Belief				4.306	0.116
No	1036(97.37%)	551(95.83%)	44(93.62%)		
Yes	28(2.63%)	24(4.17%)	3(6.38%)		
Highest Education Level				3.471	0.748
Secondary Vocational School	13(1.22%)	8(1.39%)	1(2.13%)		
Junior College	187(17.58%)	87(15.13%)	11(23.4%)		
Bachelor’s Degree	843(79.23%)	469(81.57%)	34(72.34%)		
Master’s Degree or Above	21(1.97%)	11(1.91%)	1(2.13%)		
Marital Status				38.624	< 0.001
Unmarried/Divorced/ Widowed	153(14.38%)	133(23.13%)	20(42.55%)		
Married	911(85.62%)	442(76.87%)	27(57.45%)		
Average Monthly Income				121.928	< 0.001
<3000 RMB	97(9.12%)	86(14.96%)	20(42.55%)		
3001–5000 RMB	409(38.44%)	314(54.61%)	14(29.79%)		
5001–7000 RMB	368(34.59%)	133(23.13%)	10(21.28%)		
7001–9000 RMB	143(13.44%)	30(5.22%)	2(4.26%)		
>9000 RMB	47(4.42%)	12(2.09%)	1(2.13%)		
Number of Children				25.148b	< 0.001
No Children	320(30.08%)	140(24.35%)	26(55.32%)		
One	563(52.91%)	312(54.26%)	17(36.17%)		
Two or More	181(17.01%)	123(21.39%)	4(8.51%)		
Years in Nursing Profession				13.025	0.111
0–5 Years	286(26.88%)	146(25.39%)	20(42.55%)		
6–10 Years	328(30.83%)	193(33.57%)	11(23.4%)		
11–15 Years	172(16.17%)	96(16.7%)	8(17.02%)		
16–20 Years	111(10.43%)	62(10.78%)	0(0.0%)		
20 + Years	167(15.7%)	78(13.57%)	8(17.02%)		
Years in Current Institution				10.595	0.226
0–5 Years	351(32.99%)	181(31.48%)	21(44.68%)		
6–10 Years	311(29.23%)	186(32.35%)	10(21.28%)		
11–15 Years	160(15.04%)	83(14.43%)	8(17.02%)		
16–20 Years	97(9.12%)	55(9.57%)	0(0.0%)		
20 + Years	145(13.63%)	70(12.17%)	8(17.02%)		
Department				3.184b	0.922
Internal Medicine	285(26.79%)	155(26.96%)	14(29.79%)		
Surgery	249(23.4%)	123(21.39%)	13(27.66%)		
Obstetrics and Gynecology	186(17.48%)	110(19.13%)	8(17.02%)		
Pediatrics	137(12.88%)	81(14.09%)	5(10.64%)		
Others	207(19.45%)	106(18.43%)	7(14.89%)		
Professional Title				29.853	< 0.001
No Title	47(4.42%)	23(4.0%)	6(12.77%)		
Technician Level	138(12.97%)	62(10.78%)	14(29.79%)		
Junior Professional (Initial)	500(46.99%)	301(52.35%)	13(27.66%)		
Intermediate Level	319(29.98%)	168(29.22%)	12(25.53%)		
Associate Senior or Above	60(5.64%)	21(3.65%)	2(4.26%)		
Administrative Position				35.823	< 0.005
No	842(79.14%)	521(90.61%)	41(87.23%)		
Yes	222(20.86%)	54(9.39%)	6(12.77%)		
On-the-Job Staff Status				41.426	< 0.001
No	605(56.86%)	415(72.17%)	36(76.60%)		
Yes	459(43.14%)	160(27.83%)	11(23.40%)		
Hospital Level				5.314b	0.257
Second-Class B and Below	447(42.01%)	213(37.04%)	19(40.43%)		
Third-Class B	120(11.28%)	73(12.7%)	3(6.38%)		
Third-Class A	497(46.71%)	289(50.26%)	25(53.19%)		
Average Weekly Work Hours				8.767b	0.067
≤ 40 h	519(48.78%)	240(41.74%)	20(42.55%)		
41–50 h	410(38.53%)	251(43.65%)	18(38.3%)		
> 50 h	135(12.69%)	84(14.61%)	9(19.15%)		
Monthly Night Shifts				59.943	< 0.001
≤ 7 Days	811(76.22%)	333(57.91%)	30(63.83%)		
> 7 Days	253(23.78%)	242(42.09%)	17(36.17%)		
Perceived Respect from Patients				6.717	0.152
Disrespected	56(5.26%)	38(6.61%)	2(4.26%)		
Neutral	453(42.58%)	244(42.43%)	28(59.57%)		
Respected	555(52.16%)	293(50.96%)	17(36.17%)		
Experienced Patient/Visitor Violence				45.908	< 0.001
No	633(59.49%)	245(42.61%)	19(40.43%)		
Yes	431(40.51%)	330(57.39%)	28(59.57%)		
Current Work Environment				2.123	0.713
Poor	216(20.30%)	127(22.09%)	12(25.53%)		
Moderate	542(50.94%)	278(48.35%)	24(51.06%)		
Good	306(28.76%)	170(29.57%)	11(23.40%)		
Medical Errors in Past Year				0.732	0.693
No	1038(97.56%)	557(96.87%)	46(97.87%)		
Yes	26(2.44%)	18(3.13%)	1(2.13%)		
Psychological Distress				323.654b	< 0.001
Low Distress	546(51.32%)	88(15.3%)	6(12.77%)		
Moderate Distress	295(27.73%)	151(26.26%)	14(29.79%)		
High Distress	146(13.72%)	170(29.57%)	6(12.77%)		
Severe Distress	77(7.24%)	166(28.87%)	21(44.68%)		
Work-Family Conflict				108.674b	< 0.001
Low Conflict	231(21.71%)	43(7.48%)	8(17.02%)		
Moderate Conflict	546(51.32%)	249(43.3%)	15(31.91%)		
High Conflict	287(26.97%)	283(49.22%)	24(51.06%)		
Social Support				31.597	< 0.001
Low Support	49(4.61%)	29(5.04%)	4(8.51%)		
Moderate Support	325(30.55%)	251(43.65%)	18(38.3%)		
High Support	690(64.85%)	295(51.3%)	25(53.19%)		

It is noteworthy that, for contextual comparison, the traditional Pittsburgh Sleep Quality Index (PSQI) global score cutoff of > 7 identified 27.63% (n = 466) of our sample as having poor sleep quality. This prevalence provides a benchmark against which the more nuanced, data-driven latent profile analysis (LPA) can be contrasted. The LPA approach, while identifying a smaller proportion of nurses in the ‘Low Sleep Status’ group (2.79%), further stratified the majority of nurses previously lumped into a ‘good sleeper’ category (PSQI ≤ 7) into distinct ‘High’ (63.11%) and ‘Moderate’ (34.10%) sleep quality subgroups, thereby revealing substantial heterogeneity that is obscured by the unitary PSQI cutoff.

### Univariate analysis of latent sleep status profiles

Significant differences (*P* < 0.05) were observed among the three sleep status groups across multiple sociodemographic and occupational variables, including age, marital status, average monthly income, number of children, professional rank, administrative roles, employment status (permanent vs. contract), night shift frequency, experiences of verbal/physical abuse from patients or families, psychological distress, work-family conflict, and perceived social support. Full comparative data are provided in Table 4.

### Multivariate logistic regression analysis of factors influencing sleep status groups

Using the high sleep status group as the reference, multivariate logistic regression models were constructed for the moderate and low sleep status groups. Significant predictors included marital status, average monthly income, number of children, administrative roles, employment status (permanent vs. contract), monthly night shift frequency, experiences of verbal/physical abuse from patients or families, psychological distress, and work-family conflict. In the moderate sleep status group, significant risk factors included being unmarried/divorced/widowed (OR = 2.153, 95% CI: 0.019–0.415), absence of administrative roles (OR = 1.833, 95% CI: 1.262–2.662), and contract employment (OR = 1.475, 95% CI: 1.127–1.929). Protective factors were having no children (OR = 0.339, 95% CI: 0.225–0.512), night shifts ≤ 7 days/month (OR = 0.604, 95% CI: 0.467–0.782), absence of abuse from patients/families (OR = 0.668, 95% CI: 0.524–0.852), and lower psychological distress and work-family conflict. For the low sleep status group, key risk factors were being unmarried/divorced/widowed (OR = 2.252, 95% CI: 1.015–4.996) and monthly income < 3,000 CNY (OR = 9.098, 95% CI: 1.034–80.036), while lower psychological distress and work-family conflict remained protective. Full regression results are detailed in Table 5.

**Table 5:** Multivariate logistic regression models for sleep quality latent profiles

Variable	Class 2			Class3		
	OR	95% CI	*p*	OR	95% CI	*p*
Marital Status (ref: Married)						
Unmarried/Divorced /Widowed	2.153	1.472–3.148	< 0.001	2.252	1.015–4.996	0.046
Average Monthly Income (ref: >9000RMB/month)						
<3000 RMB	2.034	0.914–4.525	0.082	9.098	1.034–80.036	0.047
3001–5000 RMB	1.826	0.875–3.810	0.109	1.695	0.196–14.622	0.631
5001–7000 RMB	0.956	0.454–2.015	0.906	1.393	0.160-12.145	0.764
7001–9000 RMB	0.520	0.227–1.190	0.122	0.663	0.055–7.982	0.746
Number of Children (ref: Two or More)						
No Children	0.339	0.225–0.512	< 0.001	2.001	0.602–6.650	0.258
One	0.847	0.616–1.165	0.307	1.537	0.494–4.780	0.458
Administrative Position(No)	1.833	1.262–2.662	0.001	0.871	0.330–2.302	0.780
On-the-Job Staff Status (No)	1.475	1.127–1.929	0.005	1.291	0.607–2.746	0.508
Monthly Night Shifts (≤ 7 Days)	0.604	0.467–0.782	< 0.001	0.847	0.434–1.652	0.625
Experienced Patient/Visitor Violence (No)	0.668	0.524–0.852	0.001	0.609	0.317–1.171	0.137
**Psychological Distress**(ref: Severe Distress)						
Low Distress	0.099	0.067–0.145	< 0.001	0.060	0.022–0.165	< 0.001
Moderate Distress	0.269	0.186–0.387	< 0.001	0.216	0.099–0.469	< 0.001
High Distress	0.555	0.379–0.813	0.002	0.170	0.064–0.456	< 0.001
Work-Family Conflict(ref: High Conflict)						
Low Conflict	0.329	0.217–0.497	< 0.001	0.657	0.266–1.623	0.362
Moderate Conflict	0.662	0.511–0.856	0.002	0.439	0.216–0.892	0.023

### Association rule analysis of clinical nurses’ sleep status

**Fig. 2:**
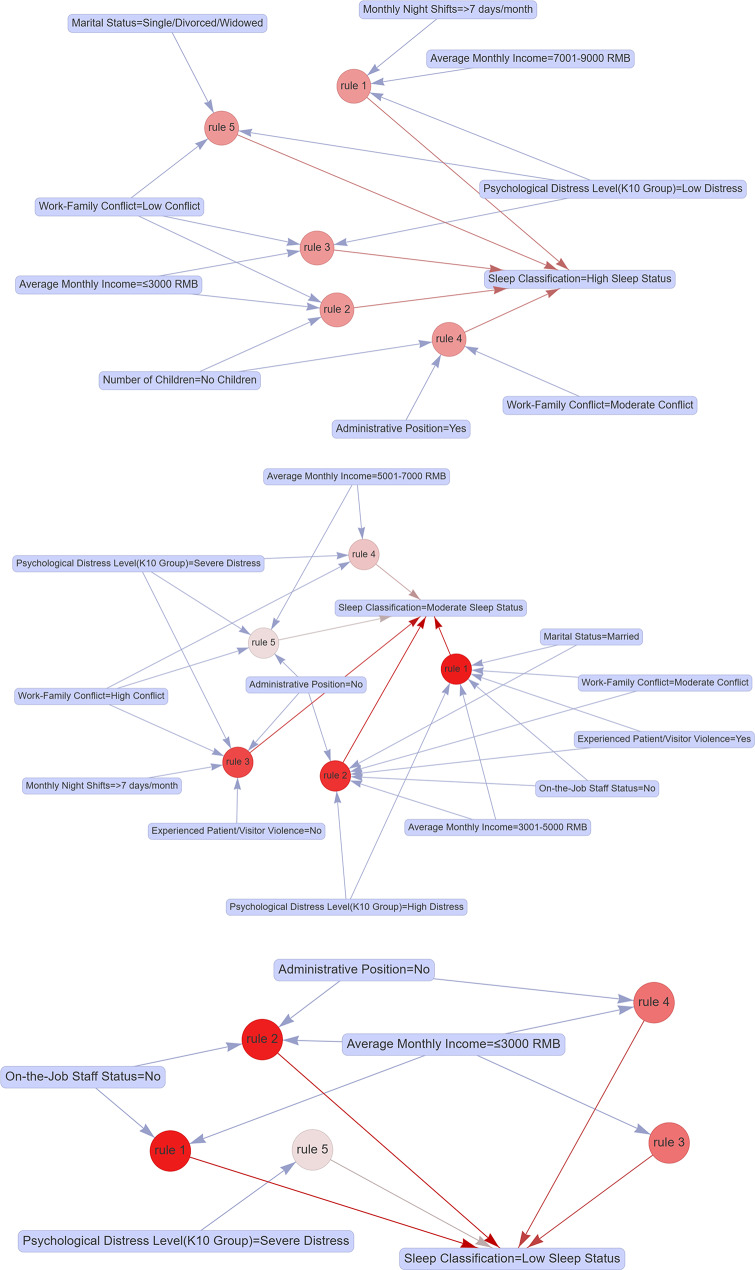
Association rule analysis of sleep status in clinical nurses

Association rule mining revealed distinct characteristic combinations for each sleep status group. The high sleep status group exhibited the highest-confidence rules, including: {monthly income = 7,001–9,000 CNY, night shifts > 7 days/month, low psychological distress}, {monthly income < 3,000 CNY, no children, low work-family conflict}, and {monthly income < 3,000 CNY, low psychological distress, low work-family conflict}. The moderate sleep status group was predominantly associated with combinations such as: {married, monthly income = 3,001–5,000 CNY, contract employment, experienced abuse, high psychological distress, moderate work-family conflict}; {married, monthly income = 3,001–5,000 CNY, no administrative role, contract employment, experienced abuse, high psychological distress, moderate work-family conflict}; and {no administrative role, night shifts > 7 days/month, no abuse, very high psychological distress, high work-family conflict}. For the low sleep status group, the most frequent rules centered on low income: {monthly income < 3,000 CNY, contract employment}, {monthly income < 3,000 CNY, no administrative role, contract employment}, and {monthly income < 3,000 CNY}. These findings highlight socioeconomic vulnerability (e.g., low income, contract employment) and occupational stressors (e.g., lack of administrative roles) as critical markers of poor sleep outcomes. Detailed rules are presented in Table 6; Fig. 2.

**Table 6:** Association rule analysis of sleep quality in clinical Nurses

Group	Order	ruler	support	confidence	coverage	lift	count
High Sleep Status	1	{Average Monthly Income = 7001–9000 RMB, Monthly Night Shifts ≥ 7 days, Psychological Distress Level (K10 Group) = Low Distress}	0.011	1.000	0.011	1.585	19
	2	{Average Monthly Income<3000 RMB, Number of Children = No Children, Work-Family Conflict = Low Conflict}	0.011	1.000	0.011	1.585	19
	3	{Average Monthly Income<3000 RMB, Psychological Distress Level (K10 Group) = Low Distress, Work-Family Conflict = Low Conflict}	0.010	1.000	0.010	1.585	17
	4	{Number of Children = No Children, Administrative Position = Yes, Work-Family Conflict = Moderate Conflict}	0.011	1.000	0.011	1.585	19
	5	{Marital Status = Unmarried/Divorced/Widowed, Psychological Distress Level (K10 Group) = Low Distress, Work-Family Conflict = Low Conflict}	0.011	1.000	0.011	1.585	19
Moderate Sleep Status	1	{Marital Status = Married, Average Monthly Income = 3001–5000 RMB, On-the-Job Staff Status = No, Experienced Patient/Visitor Violence = Yes, Psychological Distress Level (K10 Group) = High Distress, Work-Family Conflict = Moderate Conflict}	0.012	0.952	0.012	2.793	20.000
	2	{Marital Status = Married, Average Monthly Income = 3001–5000 RMB, Administrative Position = No, On-the-Job Staff Status = No, Experienced Patient/Visitor Violence = Yes, Psychological Distress Level (K10 Group) = High Distress, Work-Family Conflict = Moderate Conflict}	0.011	0.950	0.012	2.786	19.000
	3	{ Administrative Position = No, Monthly Night Shifts ≥ 7 days, Experienced Patient/Visitor Violence = No, Psychological Distress Level (K10 Group) = Severe Distress, Work-Family Conflict = High Conflict}	0.011	0.947	0.011	2.778	18.000
	4	{Average Monthly Income = 5001–7000 RMB, Psychological Distress Level (K10 Group) = Severe Distress, Work-Family Conflict = High Conflict}	0.015	0.929	0.017	2.723	26.000
	5	{Average Monthly Income = 5001–7000 RMB, Administrative Position = No, Psychological Distress Level (K10 Group) = Severe Distress, Work-Family Conflict = High Conflict}	0.014	0.923	0.015	2.707	24.000
Low Sleep Status	1	{Average Monthly Income<3000 RMB, On-the-Job Staff Status = No}	0.011	0.109	0.104	3.895	19
	2	{Average Monthly Income<3000 RMB, Administrative Position = No, On-the-Job Staff Status = No}	0.011	0.108	0.098	3.890	18
	3	{Average Monthly Income<3000 RMB}	0.012	0.099	0.120	3.534	20
	4	{Average Monthly Income<3000 RMB, Administrative Position = No}	0.011	0.098	0.114	3.531	19
	5	{Psychological Distress Level (K10 Group) = Severe Distress}	0.012	0.080	0.157	2.853	21

## Discussion

This study identified three distinct sleep profiles among 1,686 clinical nurses using latent profile analysis (LPA): high sleep status (63.11%), moderate sleep status (34.10%), and low sleep status (2.79%). This classification challenges the traditional homogeneity assumption of sleep assessment based solely on total scale scores, highlighting multidimensional differences in sleep quality.

The proportion of nurses in our study identified as having poor sleep quality via the traditional PSQI cutoff of >7 was 27.63%. This prevalence is consistent with the high burden of sleep problems documented among nurses globally, particularly in high-stress environments like China’s healthcare system [18, 19], and underscores the persistent and critical nature of addressing sleep health in this population. However, our LPA results refine this understanding. The small but severely affected ‘Low Sleep Status’ group (2.79%) captured the most vulnerable individuals, whom the PSQI cutoff would have included within the broader 27.63%. More importantly, LPA succeeded in segmenting the remaining population into meaningful subgroups, revealing that a substantial proportion (34.10%) experience moderate but distinct sleep challenges that warrant targeted attention. This demonstrates that LPA moves beyond a simple ‘good-poor’ dichotomy to uncover the underlying heterogeneity and complex interplay of factors affecting nurses’ sleep.

Rather than seeking a universal “natural law” of sleep determinants, our findings provide a nuanced, data-driven case study that captures the complex interplay of factors within a specific socio-cultural and occupational context. In line with a “coherence” perspective on scientific validity, sleep quality is best understood not as a decontextualized biological absolute, but as a construct shaped by an individual’s specific circumstances, including their work environment, socioeconomic resources, and psychological state. The latent profile analysis employed here is particularly suited to this view, as it does not presume population homogeneity but actively identifies meaningful subgroups that may be obscured by aggregate-level analysis. The three profiles we identified—each with its own configuration of risk and protective factors—serve as a testament to this underlying heterogeneity. Thus, the value of this study lies in its contribution to a growing body of literature; its patterns and associations await confirmation and comparison through future replications in diverse settings to build a more coherent, generalizable understanding of nurses’ sleep health.

The low sleep status group (*n* = 47) exhibited marked vulnerability: 42.55% were unmarried/divorced/widowed, 42.55% had a monthly income ≤ 3,000 CNY, 76.60% held non-permanent employment status, and 44.68% reported severe psychological distress—all significantly higher than other groups (*p* < 0.001). Notably, this group showed greater variability in sleep efficiency (mean = 0.53, SD = 0.86) and daytime dysfunction (mean = 1.20, SD = 0.91), indicating pronounced internal heterogeneity. These findings suggest that the convergence of occupational instability (non-permanent status), financial strain (low income), and inadequate social support (unmarried status) can converge to lowers stress tolerance thresholds, potentially explaining why minor stressors may trigger systemic dysregulation [20]. McEwen and Karatsoreos’ allostatic load theory provides a plausible biological framework for these patterns: chronic stress disrupts cortisol rhythms via HPA axis hyperactivity, suppresses melatonin secretion, and induces neuroinflammation, ultimately destabilizing sleep homeostasis [21]. In contrast, the high sleep status group demonstrated protective characteristics: 85.62% were married, 43.14% held permanent employment, and most had moderate incomes. This group exhibited significantly lower psychological distress, which aligns with the social support buffering model—whereby stable marital relationships may reduce sympathetic nervous system activity through emotional and economic support [22], and permanent employment could help to mitigates shiftwork-induced circadian disruptions [23].

Univariate and multivariate logistic regression analyses identified core differentiating variables across sleep subgroups: marital status, economic income, occupational stability, night shift frequency (*p* < 0.001), and psychological state (*p* < 0.001) collectively formed a multidimensional predictive network for sleep quality. Specifically, unmarried/divorced/widowed nurses had 2.153-fold (95% CI: 1.472–3.148) and 2.252-fold (95% CI: 1.015–4.996) higher risks of belonging to the moderate and low sleep groups, respectively, compared to married individuals. This aligns with prior research indicating that the absence of spousal emotional support and economic collaboration depletes stress-coping resources, exacerbating sympathetic nervous system activity and impairing sleep maintenance mechanisms [22].The impact of financial strain on sleep exhibited a dose-response relationship: nurses with monthly incomes ≤ 3,000 CNY faced a 9.098-fold (95% CI: 1.034–80.036) increased risk of entering the low sleep group compared to high-income counterparts (≥ 9,000 CNY), corroborating findings by Goh et al. that economic hardship amplifies anxiety and reduces sleep efficiency via hyperactivity in prefrontal-limbic neural circuits [24]. Occupational stressors demonstrated significant interactive effects: non-permanent employment status combined with frequent night shifts (>7 days/month) elevated sleep disorder risk by 2.7-fold. Notably, psychosocial factors revealed clinically meaningful gradients: nurses with low psychological distress had 90.1% (OR = 0.099, 95% CI: 0.067–0.145) and 94.0% (OR = 0.060, 95% CI: 0.022–0.165) lower risks of moderate and low sleep group membership, respectively, compared to those with severe distress. Additionally, the moderating role of work-family conflict in the moderate sleep group highlighted a unique dilemma among married nurses: while partial balancing of family responsibilities and occupational demands may transiently alleviate stress, it fails to fully counteract sleep quality deterioration. Sustained depletion of psychological resources under dual work-family role conflicts weakens stress-buffering capacity, ultimately leading to sleep fragmentation [25]. These findings underscore the need for interventions targeting married nurses to prioritize flexible scheduling and childcare support, fostering dynamic equilibrium of stressors.

The application of the Apriori algorithm (minimum support = 0.01, confidence = 0.90) uncovered distinct characteristic combinations and interaction mechanisms across nurse sleep subgroups. For nurses in the low sleep status group, the core association rule {monthly income ≤ 3,000 CNY + non-permanent employment status} (support = 1.1%, lift = 3.89) highlighted a synergistic mechanism wherein financial precarity and occupational instability jointly accounted for 76.60% of sleep disorder risk. This underscores the compounding effects of socioeconomic vulnerability and systemic employment inequities. In the moderate sleep status group, the rule {married + monthly income 3,001–5,000 CNY + non-permanent employment + experienced verbal/physical abuse + high psychological distress} (support = 1.1%, lift = 2.78) revealed a multidimensional stress burden among married nurses. Despite potential emotional support from marital relationships, the interplay of employment insecurity (non-permanent status) and workplace violence effectively neutralized the protective effects of familial bonds, suggesting that structural stressors dominate over individual resilience factors in this subgroup. Conversely, the high sleep status group exhibited adaptive resilience through rules such as {monthly income ≥ 7,001 CNY + night shifts > 7 days/month + low psychological distress} (support = 1.2%, lift = 2.79). Here, financial security mitigated anxiety-related neurophysiological dysregulation, while low psychological distress buffered the physiological impacts of frequent night shifts, such as melatonin suppression, demonstrating how resource abundance and psychological stability interact to sustain sleep health.

Building on these findings, which unveil distinct risk configurations through both LPA and association rule mining, a stratified intervention strategy is imperative. Moving beyond one-size-fits-all approaches, our data advocate for precision interventions that target the specific vulnerabilities of each subgroup, with an emphasis on pragmatism within the current healthcare system. For the vulnerable Low-Sleep Subgroup, interventions must address the synergistic threat of socioeconomic precarity. System-level advocacy to improve income stability and contract conditions for non-permanent nurses is fundamental, as financial strain is a paramount risk factor [26, 27]. At the organizational level, guaranteed access to low-threshold psychological first aid [28, 29] and stringent enforcement of rest periods after night shifts [30] are critical, feasible steps to prevent allostatic overload. For the Moderate-Sleep Subgroup, characterized by the interplay of work-family strain and occupational stressors, targeted support is key. Hospital administrators should prioritize the implementation of flexible scheduling where feasible [31], robust violence de-escalation training [32], and the provision of affordable childcare support. These measures directly address the multidimensional stress burden that erodes sleep quality in this large segment of the nursing workforce. For the resilient High-Sleep Subgroup, the focus should shift to preservation. Efforts here can center on reinforcing existing protective factors through continued professional respect, access to mindfulness resources to maintain low psychological distress, and upholding fair shift schedules to sustain their adaptive resilience [33].

Despite revealing heterogeneous sleep characteristics and multidimensional mechanisms among nurses through latent profile analysis and association rule mining, this study has several limitations. This study has several limitations, which should be taken into account when interpreting the study findings. First, regarding the study design, the cross-sectional nature of our data precludes the establishment of a causal relationship between the identified factors and sleep profiles. Second, in terms of measurement, our reliance on self-reported questionnaires may introduce recall bias and social desirability bias. The lack of objective sleep measurements and data on key potential confounders—such as specific clinical conditions and napping behavior—limits the comprehensiveness and clinical specificity of our assessments. Third, regarding sample representativeness, our data were derived from one province in China, which may affect the generalizability of the study findings to nurses in other healthcare systems and cultural contexts. Furthermore, the small size of the subgroup with poor sleep quality (*n* = 47) may compromise the statistical stability of its associated characteristics. Additionally, due to the exclusion of nurses who had left the profession due to severe sleep issues, this study may be subject to survivorship bias. Finally, from a methodological and epistemological perspective, although our analytical approach is robust in identifying data-driven subgroups, it operates within a framework that seeks a coherent internal structure. This inherently embeds assumptions about the divisibility of the population and fails to incorporate organizational-level influences, potentially overlooking broader contextual effects. Future studies should directly address these limitations. Longitudinal cohorts are essential for validating sleep profiles and establishing causal relationships. Integrating multimodal assessments—including objective sleep measurements and clinical evaluations—will provide a more robust understanding of sleep architecture and underlying mechanisms. Crucially, incorporating qualitative methods can help verify whether the identified statistical profiles resonate with nurses’ subjective lived experiences. Finally, studies need to be conducted to test the feasibility and effectiveness of the proposed targeted interventions in diverse healthcare settings.

Future research should address these gaps by expanding to multiregional, large-scale cohorts to enhance external validity and subtype identification accuracy. Longitudinal designs are needed to track dynamic interactions between sleep quality trajectories and occupational health outcomes, particularly in high-stress healthcare environments. Integrating multimodal assessments—including actigraphy, PSG, and neuroendocrine biomarkers (e.g., cortisol, melatonin)—would strengthen the mechanistic understanding of sleep disturbances and validate intervention efficacy. Implementation studies testing the proposed “institutional safeguards–precision interventions” framework across diverse clinical settings are critical to refining strategies for real-world applicability. Collectively, these advancements will provide robust evidence to establish a comprehensive “prevention-intervention-follow-up” continuum in nurse sleep health management.

## Data Availability

Due to the regulatory requirements of the Ethics Committee, the supporting data of this study are not available to the public, but can be obtained upon reasonable request from the corresponding author with the consent of the Ethics Committee of the School of Public Health, Shandong University.
